# Oat Seedlings Extract Inhibits RANKL-Induced c-Fos/NFATc1 Transcription Factors in the Early Stage of Osteoclast Differentiation

**DOI:** 10.1155/2022/5372459

**Published:** 2022-09-23

**Authors:** Ji Yeong Yang, Shin-Hye Kim, HanGyeol Lee, Kwang-Sik Lee, Seung-Yeob Song, Mi Ja Lee, Hyun Young Kim, So-An Lim, Kie-In Park, Sik-Won Choi, Woo Duck Seo

**Affiliations:** ^1^Crop Foundation Research Division, National Institute of Crop Science (NICS), Rural Development Administration (RDA), Wanju 55365, Republic of Korea; ^2^Forest Biomaterials Research Center, National Institute of Forest Science (NIFoS), Korea Forest Service (KFS), Jinju 52817, Republic of Korea; ^3^Department of Biological Sciences, College of Natural Science, Jeonbuk National University, Jeonju 54896, Republic of Korea; ^4^Fruit Research Division, National Institute of Horticultural and Herbal Science (NIHHS), Rural Development Administration (RDA), Wanju 55365, Republic of Korea; ^5^Department of Forest Environmental Resources, College of Agriculture and Life Science, Gyeongsang National University, Jinju 52828, Republic of Korea

## Abstract

Osteoporosis is a common disease that increases the risk of fractures due to decreased bone density and weakens the bone microstructure. Preventing and diagnosing osteoporosis using the available drugs can be a costly affair with possible side effects. Therefore, natural product-derived therapeutics are promising alternatives. Our study demonstrated that the oat seedlings' extract (OSE) inhibited the receptor activator of the nuclear factor *κ*B ligand (RANKL)-induced osteoclastogenesis from the bone marrow-derived macrophages (BMMs). The OSE treatment significantly attenuated the RANKL-mediated induction of the tartrate-resistant acid phosphatase (TRAP) activity as well as the number of TRAP-positive (TRAP+) multinucleated cells (MNCs) counted through the TRAP staining in a dose-dependent manner. It was also confirmed that the OSE suppressed the formation of the TRAP + MNCs in the early stage of differentiation and not in the middle and late stages. The results of the real-time quantitative polymerase chain reaction (qPCR) and the western blotting showed that the OSE dramatically inhibited the mRNA and protein expressions of the osteoclastogenesis-mediated transcription factors such as the c-Fos and the nuclear factor-activated T cells c1 (NFATc1). In addition, the OSE strongly attenuated the mRNA induction of the c-Fos/NFATc1-dependent molecules such as the *TRAP*, the osteoclast-associatedimmunoglobulin-like receptor (*OSCAR*), the dendritic cell-specific transmembrane protein (*DC-STAMP*), and the *cathepsin K*. These results suggest that the naturally derived OSE may be useful for preventing bone diseases.

## 1. Introduction

Osteoporosis is a bone-related disease in which the bone mass, structure, and quality deteriorate, resulting in decreased bone strength and increased risk of fracture. A bone density of 2.5 standard deviations below that of the young, white female population defines osteoporosis [1]. In developed countries such as North America, Europe, Japan, and Australia, the prevalence of hip and spine osteoporosis is 1–8% and 9–38% in men and women, respectively [2]. Osteoporosis is typically seen in menopausal women, and the main associated factor is reduced estrogen levels after menopause. In men, bone mass loss occurs due to decreased testosterone levels [3].

Bone metabolism comprises the processes of bone production by osteoblasts and bone degradation by osteoclasts. Increased osteoclasts cause an excessive bone breakdown, which in turn leads to bone metabolic disorder. Osteoclast differentiation is mainly mediated by the receptor activator of the nuclear factor *κ*B ligand (RANKL) [4]. The RANKL activates the nuclear factor *κ*B (NF-*κ*B) through multiple pathways in the initial stage of osteoclast differentiation. Interaction of the RANKL with its receptor RANK induces RANK binding to a member of the tumor necrosis factor (TNF) receptor-associated factor (TRAF) family. TRAF6 plays the most significant role in osteoclast differentiation and activates the NF-*κ*B downstream of the TRAF6-RANK complex [5]. The activated NF-*κ*B activates the c-Fos, which binds to the nuclear factor-activated T cells c1 (NFATc1) and inhibits the NFATc1 receptor in the early-to-middle stages [6]. In the middle-to-late stages, increased NFATc1 upregulates the specific gene expressions such as the dendritic cell-specific transmembrane protein (DC-STAMP), cathepsin-K, and the osteoclast-associatedimmunoglobulin-like receptor (OSCAR), which induces osteoclast differentiation, cell fusion, and maturation [7–10].

For the treatment of osteoporosis, various therapeutic agents such as calcium, vitamin D, selective estrogen receptor modulator, bisphosphonate, parathyroid hormone, and anti-RANKL antibody have been used [11]. However, these therapeutic agents induce side effects, especially on prolonged usage [12]. For example, treatment with bisphosphonate can cause osteonecrosis of the jaw, acute renal failure, or gastrointestinal disorders; calcium causes gastrointestinal tract disorders or hypercalcemia; and hormonal therapy leads to stroke and pulmonary embolism. Due to the chronic systemic nature of the disease, the osteoporosis treatment course is long, and the accompanying side effects place a great burden on patients, especially elderly patients. Recently, a complex method involving anabolic treatment followed by sequential treatment with an antiabsorption agent has been suggested [13]. Candidates suitable for this treatment are commonly available in the vicinity. In addition, treatment using natural medicinal products with few or no side effects from ingestion can be a good substitute.

Natural products have long been consumed by mankind as ancient medicines. In EU countries, medicinal products extracted from natural substances are called herbal medicinal products. In the United States, natural medicines are approved as botanical drug products. Germinated grains have received considerable attention to be consumed as healthy functional foods and natural medicines over the past decade. Grains are mainly eaten as seeds; however, there have been various reports on increased nutritional status and secondary metabolites in grain seedlings [14, 15]. For example, oat seedlings have significantly increased protein content, beta-glucan levels, free amino acids, and phenolic compounds compared to that in seeds and contain various secondary metabolites [16, 17]. Especially, we have previously identified 11 polyphenolic compounds of oat seedlings and then selected oats seedlings cultivars with high metabolite content [18]. Also, we have studied the effects of avena furanol and diosgenin, secondary metabolites of sprouted oats, on osteoclast differentiation [19]. Herein, we investigated the inhibitory effect of an oat seedlings' extract (OSE) on RANKL-mediated osteoclast differentiation and the underlying inhibition mechanism to demonstrate the benefits of oat seedlings in preventing and treating osteoporosis.

## 2. Materials and Methods

### 2.1. Oat Seedlings' Cultivation and Preparation of OSE

Oat seedlings (cultivar: Gwanghan) were grown at the National Institute of Crop Science (NICS), the Rural Development Administration (RDA), Korea. The growth conditions were carried out as we reported previously [18]. A plant culture chamber using soil was used, and the temperature was maintained at 18–20°C and humidity at 60–70%. The light condition was long day, with 16 h light/8 h dark conversion, and the illumination intensity was 3300–5500 lux. The seedlings were harvested on the 10th day and dried in an oven at 50°C for 24 h. Subsequently, the dried seedlings were extracted by adding 50% fermented ethanol (Duksan General Science, Seoul, Korea) in 10 folds (w/v) and stirring for 10 h. The extract was concentrated using a stirring concentrator (EYELA, Tokyo, Japan) to obtain a dry powder. The chromatogram of OSE is shown in Supplementary Figure 1.

### 2.2. Preparation of Preosteoclasts

Experiments for the preparation of preosteoclasts were performed as previously described [20]. Briefly, bone marrow cells (BMCs) were isolated from the femurs and tibias of the imprinting control region (ICR) mice (Damul Science Co., Deajeon, Korea) using *α*-minimal essential medium (*α*-MEM) (Invitrogen Life Technology, CA, USA) supplemented with 1% penicillin/streptomycin (Invitrogen Life Technology). Then, the BMCs were cultured in *α*-MEM supplemented with 10% foetal bovine serum (FBS) (Invitrogen Life Technology), 1% penicillin/streptomycin, and 10 ng/mL macrophage-colony stimulating factor (M-CSF) (R&D systems, MN, USA). After one day, the nonadherent BMCs were plated on Petri dishes and cultured with a medium containing 30 ng/mL M-CSF. After three days, the adherent cells were used as bone marrow-derived macrophages (BMMs).

All animal procedures were conducted in strict accordance with the recommendations of the standard protocol for Animal Study of Gangnam Severance Hospital Biomedical Center (Permit No. 2016-0238). The experimental protocol (ID No. 0238) was approved by the Institutional Animal Care and Use Committee (IACUC) at Yonsei University College of Medicine. Every effort was made to minimize the number of animals and their suffering, stress, and discomfort.

### 2.3. Osteoclast Differentiation

The BMMs were maintained in *α*-MEM containing 10% FBS and 1% penicillin/streptomycin in 5% CO_2_ at 37°C with medium changes every three days. To generate osteoclasts from BMMs, cells were seeded on a 96-well tissue culture plate at a density of 1 × 10^4^ cells/well or on a 6-well tissue culture plate at a density of 3 × 10^5^ cells/well, followed by treatment with 30 ng/mL M-CSF and 10 ng/mL RANKL (R&D Systems) for four days.

### 2.4. Tartrate-Resistant Acid Phosphatase (TRAP) Staining Assay

BMMs were incubated with the M-CSF (30 ng/mL) and the RANKL (10 ng/mL) in the presence of various concentrations of the OSE. A group treated only with an equal amount of vehicle (solvent) without the extract was set as a control group. After 4 days, the cell monolayer was gently washed twice with the phosphate buffer saline (PBS), fixed with 10% formalin for 10 min, and then permeabilized with 0.1% Triton X-100 for 10 min. The TRAP solution (Sigma-Aldrich) was added to the well and subsequently incubated for 10 min at 37°C. After air-drying, the TRAP-positive (TRAP+) multinucleated cells (MNCs) (containing three to ten nuclei) were counted as mature osteoclasts.

To measure the TRAP activity, the MNCs were fixed, permeabilized, and then treated with the TRAP buffer (100 mM sodium citrate (pH 5.0) and 50 mM sodium tartrate) containing 3 mM *p*-nitrophenyl phosphate (Sigma-Aldrich) at 37°C for 5 min. The reaction was stopped by adding an equal volume of 0.1 N NaOH, and the absorbance was then measured at 405 nm by using a SpectraMax M5 fluorescence spectrophotometer (Molecular Devices, CA, USA).

### 2.5. Cytotoxicity Assay

The BMMs were seeded at a density of 1 × 10^4^ cells/100 *μ*L on a 96-well plate and treated with the M-CSF (30 ng/mL) and the OSE (3–100 *μ*g/mL) for three days. To assess cell viability, the Cell Counting Kit-8 reagent (Dojindo Molecular Technologies, MD, USA) was added directly to each well of the plate and then incubated at 37°C. After 1 h, the viable cell number was determined by measuring the optical density at 450 nm on the spectrophotometer.

### 2.6. F-Actin Ring Staining

Preosteoclasts were cultured in the presence of the M-CSF (30 ng/mL) and the RANKL (10 ng/mL) on clear, black glass plates. After three days, the OSE or the DMSO (vehicle) was treated for one day. At the end of the incubation, the cells were fixed, permeabilized as mentioned above, and then stained with the phalloidin-FITC (Sigma-Aldrich) under dark conditions for 10 min. The actin rings of mature osteoclasts were photographed under a fluorescence microscope (10x magnification).

### 2.7. Bone-Pit Formation

Mature osteoclasts were obtained by isolating osteoblasts from the calvariae of newborn mice through serial digestion with 0.1% collagenase (Gibco, Paisley, UK), as previously described [21]. On a collagen-coated plate, osteoblasts and BMMs were seeded at a density of 3.5 × 10^5^ cells/well and 1 × 10^6^ cells/well, respectively, and then cocultured in a complete medium containing 1*α*, 25-dihydroxy, vitamin D3, and prostaglandin *E*2 with medium changes every three days. After six days, the cells were detached and replated on a bone biomimetic synthetic surface (Corning, NY, USA) of a 24-well tissue culture plate for 1 h. Thereafter, the cells were treated with the RANKL (10 ng/mL) and the OSE for one day. To observe the resorption pits, the slides were washed twice with PBS and treated with 5% sodium hypochlorite solution for 5 min. The plate was washed again with PBS, air-dried, and then observed under a light microscope. The resorbed areas were quantified by using the ImageJ software [21].

### 2.8. Real-Time PCR

The total RNA from the cultured BMMs was extracted with the TRIzol reagent (Invitrogen) according to the manufacturer's recommended protocol. All experiments were conducted only with high-quality RNA (A260/A280 ratio of 1.8–2.0 and A260/A230 ratio of 2.0–2.2). Reverse transcription was performed with 1 *μ*g of RNA and RevertAid First Strand cDNA Synthesis Kit (Thermo Fisher Scientific, MA, USA) according to the manufacturer's protocol. The cDNA was amplified by using Applied Biosystems Power-Up SYBR green PCR master mix (Thermo Fisher Scientific) and quantified by using Quantstudio®5 Real-Time PCR (Thermo Fisher Scientific). Primers were used as previously reported [17], and the genes were normalized to encoding glyceraldehyde 3-phosphate dehydrogenase (*GAPDH*). All real-time PCR reactions were performed at least three times, and all data were analyzed by using the 2^−ΔΔCt^ method [22].

### 2.9. Western Blotting

Cells were lysed in radioimmunoprecipitation assay (RIPA) lysis buffer (Cell Signaling Technology, MA, USA) supplemented with protease inhibitors (Basel, Switzerland) on ice. Lysates were centrifuged for 15 min at 13,000 rpm and the supernatant was collected. The amount of protein in each supernatant was quantified using the detergent-compatible (DC) protein assay kit (Bio-Rad, CA, USA). The proteins separated by gel electrophoresis were transferred onto a polyvinylidene difluoride membrane (PVDF). The membrane was blocked for 1 h in 5% skim milk, followed by overnight incubation at 4°C with primary antibodies in 1% bovine serum albumin (BSA). Thereafter, the membrane was incubated with the secondary antibodies in 5% skim milk at 25°C for 2 h. To detect protein expression, the membrane was developed by using Super Signal West Femto Maximum Sensitivity Substrate (Thermo Fisher Scientific) and visualized by using a Chemidoc XRS + imaging system (Bio-Rad).

### 2.10. Statistical Analyses

Each experiment was repeated three to five times. All quantitative values are expressed as the mean ± standard deviation (SD) and compared using Student's *t* test. A value of *p* < 0.05 was considered statistically significant.

## 3. Results

### 3.1. OSE Inhibits RANKL-Induced Osteoclastogenesis

**Figure 1:**
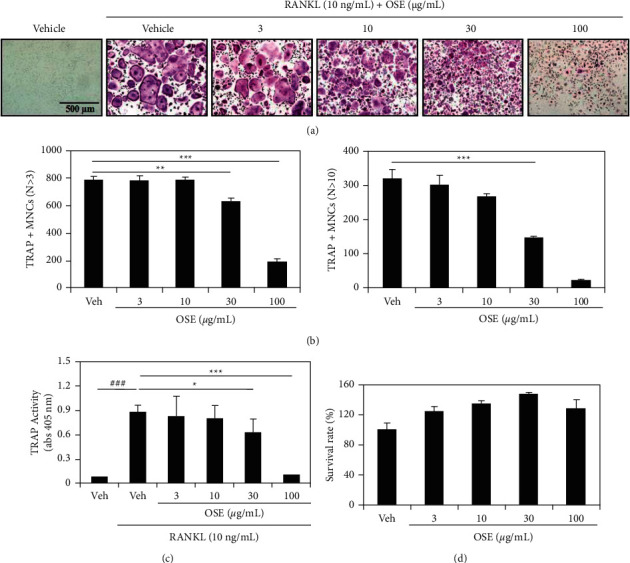
Effect of the oat seedlings' extract (OSE) on osteoclast differentiation. Bone marrow-derived macrophages (BMMs) were cultured in the presence of the receptor activator of nuclear factor *κ*B ligand (RANKL; 10 ng/mL) and the macrophage-colony stimulating factor (M-CSF; 30 ng/mL) for four days and treated with vehicle or the indicated concentration of OSE. (a) Visualization of multinucleated cells (MNCs) by using the tartrate-resistant acid phosphatase (TRAP) staining (10x magnification). (b) Counting of the TRAP-positive MNCs as osteoclasts. (c) Estimation of the TRAP activity at 405 nm. (d) Evaluation of cytotoxicity of the OSE by using the Cell Counting Kit-8. Data are expressed as mean ± SD. ^*∗*^*p* < 0.05, ^*∗∗*^*p* < 0.01, ^*∗∗∗*^*p* < 0.001 (versus the RANKL-treated control); ^###^*p* < 0.001 (versus the control).

To determine whether the OSE regulates the RANKL-mediated osteoclast differentiation, mouse BMMs were incubated with the M-CSF and the RANKL in the presence of various concentrations of the OSE for four days. In the control group (vehicle treatment), the formation of numerous TRAP + MNCs was induced by the RANKL (Figure 1(a)). On the contrary, the OSE treatment significantly attenuated this induction in a dose-dependent manner (Figure 1(a)). Furthermore, the anti-osteoclast formation activity of OSE was confirmed by counting the number of TRAP + MNCs during the differentiation (nuclei ≥3; Figure 1(b) left graph) or maturation (nuclei ≥10; Figure 1(b) right graph) stage. Consistent with this result, the OSE dramatically inhibited the TRAP activity (Figure 1(c)). However, the presence of OSE did not affect the survival of BMMs (Figure 1(d)), indicating that its inhibitory effect on osteoclastogenesis was not due to cytotoxicity. These results showed that the OSE drastically inhibited the RANKL-induced osteoclast formation without apparent cytotoxicity during osteoclast differentiation.

### 3.2. OSE Inhibits Osteoclast Differentiation in the Initial Stages

**Figure 2:**
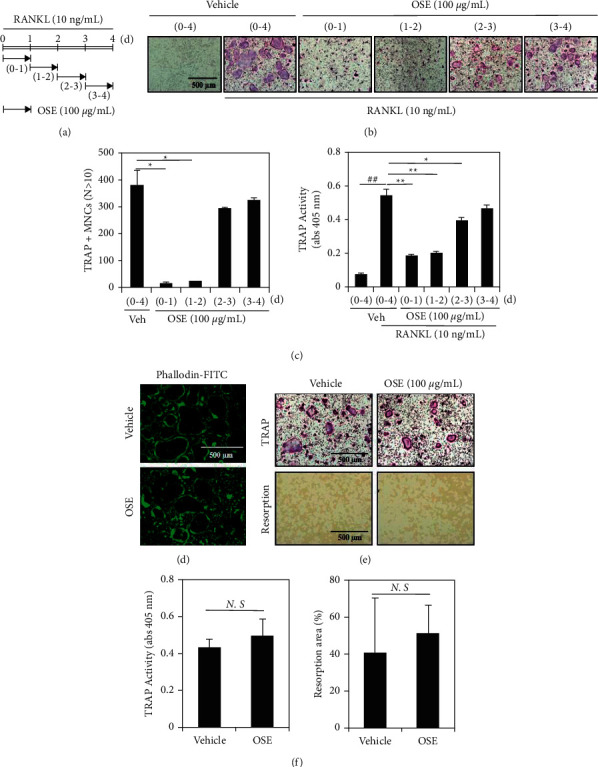
Inhibitory effect of osteoclast differentiation in the early stages in the presence of oat seedlings' extract (OSE). (a) Representation of the exposure schedule for OSE (100 *μ*g/mL) treatment of the bone marrow-derived macrophages (BMMs) for various time periods (the indicated black arrow) in the presence of macrophage-colony stimulating factor (M-CSF; 30 ng/mL) and the receptor activator of nuclear factor *κ*B ligand (RANKL; 10 ng/mL). (b) Formation of the tartrate-resistant acid phosphatase-positive (TRAP+) multinucleated cells (MNCs) photographed under a light microscope (10x magnification). (c) Counting of the TRAP + MNCs (left panel) and the TRAP activity estimation (right panel). (d) Staining of the actin rings of mature osteoclast with the phalloidin-FITC. (e) Visualization of the TRAP + MNCs (top images) and resorption areas (bottom images) under a light microscope (10x magnification). The resorption areas were photographed by removing the cells. Results of one representative experiment out of three independent experiments yielding similar results are shown. (f) Measurement of the TRAP activity during osteoclast apoptosis on the slides (left panel) and quantification of the resorptive areas (%) by using the ImageJ program (right panel). N.S, not significant; ^##^*p* < 0.01 (versus the control); ^*∗*^*p* < 0.05, ^*∗∗*^*p* < 0.01 (versus the RANKL-treated control).

To better understand how the OSE inhibits osteoclast differentiation, we investigated the anti-osteoclastogenic activity of the OSE during differentiation stages according to the exposure schedule shown in Figure 2(a). Treatment with the OSE (100 *μ*g/mL) for 24 h in the early stages of differentiation (0 to 1 and 1 to 2 days) markedly inhibited the formation of the TRAP + MNCs; however, in the middle to late stages (2 to 3 and 3 to 4 days), the OSE did not affect the formation of osteoclasts (Figure 2(b)). Additionally, the number of TRAP + MNCs (nuclei ≥10) was decreased (Figure 2(c); left graph), and the TRAP activity was significantly reduced (Figure 2(c); right graph) in the initial stages (0 to 1 and 1 to 2 days) after the OSE treatment.

To further clarify the inhibitory efficacy of the OSE during the initial stage of osteoclastogenesis, we investigated whether the fusion and function of osteoclasts in the middle to late stages were affected by the OSE treatment. Actin ring formation is the defining characteristic of osteoclast fusion in the middle stages of osteoclast differentiation. The incubation of preosteoclasts with 100 *μ*g/mL OSE for one day did not affect the formation of giant osteoclasts with actin rings, as shown by fluorescence-conjugated phalloidin staining in Figure 2(d). In addition, the bone pit formation (Figure 2(e)) and the TRAP activity (Figure 2(f)), characteristically defined by respective functional activation and apoptosis of osteoclasts, were not significantly altered in mature osteoclasts after the OSE treatment for one day. These results suggested that the anti-osteoclastogenic activity of the OSE could be specifically observed in the early stages of osteoclast differentiation.

### 3.3. OSE Does Not Contribute to the RANKL-Mediated Early Signaling Pathway in Osteoclastogenesis

**Figure 3:**
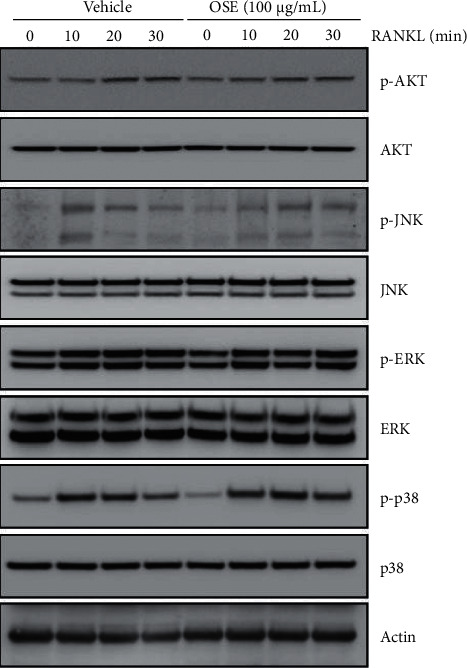
Effect of the oat seedlings' extract (OSE) on signaling molecules involved in early osteoclast differentiation. Bone marrow-derived macrophages (BMMs) were pre-treated with the OSE (100 *μ*g/mL) or vehicle for 1 h prior to the receptor activator of nuclear factor *κ*B ligand (RANKL) stimulation (10 ng/mL) at the indicated time periods. Western blotting analysis of phosphorylated and total AKT, JNK, ERK, and p38 was performed. Actin was used as a loading control.

To better understand how the OSE treatment inhibits the initial osteoclast differentiation, we explored its effects on the RANKL-mediated early signaling pathways. Interestingly, the RANKL stimulated the phosphorylation of the AKT and MAP kinases such as JNK, ERK, and p38; however, the OSE treatment had no significant effect on the phosphorylation of these factors (Figure 3). These results indicated that the OSE did not affect the early signaling molecules involved in the RANKL-induced osteoclast differentiation pathways.

### 3.4. OSE Attenuates Osteoclastogenesis-Related Molecules by Blocking c-Fos/NFATc1 Transcription Factors

**Figure 4:**
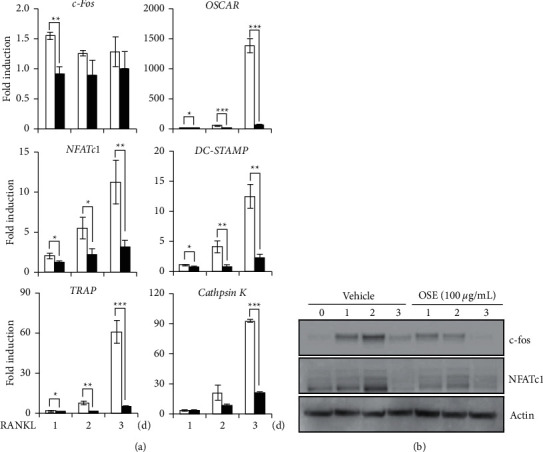
Effect of the oat seedlings' extract (OSE) treatment on osteoclastogenesis-mediated transcription factors. (a) The mRNA expression levels of the *c-*Fos, the nuclear factor-activated T cells c1 (*NFATc1*), and the c-Fos/NFATc1-dependent molecules (tartrate-resistant acid phosphatase (TRAP), osteoclast-associatedimmunoglobulin-like receptor (OSCAR), the dendritic cell-specific transmembrane protein (DC-STAMP), and the cathepsin K) as evaluated by real-time PCR. Glyceraldehyde 3-phosphate dehydrogenase (GAPDH) was used as the internal control. Bone marrow-derived macrophages (BMMs) were stimulated with the receptor activator of nuclear factor *κ*B ligand (RANKL; 10 ng/mL) and the macrophage-colony stimulating factor (M-CSF; 30 ng/mL) in the absence or presence of OSE (100 *μ*g/mL) for the indicated times. ^*∗*^*p* < 0.05; ^*∗∗*^*p* < 0.01; ^*∗∗∗*^*p* < 0.001 (versus the vehicle control). (b) Analysis of expression of the RANKL-induced mediators by using western blot analysis after the OSE and vehicle treatment. Actin was used as the internal control. Data are representative of at least three independent experiments.

To gain insights into the suppressive mechanism of the OSE in the early stages of osteoclast differentiation, we analyzed the osteoclastogenesis-mediated genes, including the RANKL-related transcription factors. As shown in Figure 4(a), the mRNA expressions of the c-Fos and the NFATc1 osteoclastogenesis-mediated transcription factors were induced by the RANKL. However, the induction was dramatically downregulated in the presence of the OSE. The western blot analysis also revealed that the OSE exposure decreased the RANKL-mediated induction of c-Fos and NFATc1 proteins (Figure 4(b)). In addition, it was confirmed that the mRNA levels of TRAP, OSCAR, DC-STAMP, and cathepsin K, c-Fos/NFATc1-dependent molecules, were decreased by the OSE. These findings demonstrated that the inhibitory effect of the OSE in the early stage of osteoclast differentiation could arise from its potential to inhibit the expression of the c-Fos/NFATc1, the master transcription factors required for osteoclastogenesis.

## 4. Discussion

Bone homeostasis depends on the balance between osteoclast-mediated bone resorption and osteoblast-mediated bone matrix formation and is affected by complex regulatory steps. When the rate of bone resorption is faster than that of bone formation, bone homeostasis collapses, and osteoporosis occurs [23]. The primary treatment for osteoporosis consists of inhibiting bone resorption by inhibiting osteoclast differentiation.

The RANKL, expressed in osteoblasts or their precursors, binds to the RANK on the surface of osteoclasts or their precursors and is an important regulator that regulates osteoclast activity and differentiation, including the M-CSF. After the MAPK signaling pathway is activated and transcription factors such as c-FOS, NFATc1, and NF-*κ*B are expressed, bone destruction occurs by the expression of enzymes such as TRAP and cathepsin K [24–26]. In our study, the artificial RANKL and the M-CSF treatment induced the TRAP + MNC formation, which was significantly reduced by the OSE. The TRAP activity was also significantly reduced (Figure 1).

As mentioned previously, osteoclasts induced by the RANKL and the M-CSF undergo differentiation through the initial MAPK pathway. In our experiment, it was confirmed that the OSE suppressed the formation of the TRAP + MNCs between 0 and 2 days during osteoclast differentiation (Figure 2). Therefore, the MAPK pathway can be considered a candidate pathway affected by the OSE. The MAPK regulates cell proliferation. Several factors, such as the ERK triggers the AP1 activity to induce the cyclin D1 induction, the ERK1/2 is related to cell survival, and the JNK and the p38 induce apoptosis [27–29]. However, we could not identify any associations between the OSE treatment and MAPKs, including p-AKT and p-JNK (Figure 3).

The influence of the OSE is induced after the RANKL and the M-CSF treatment, and in the process leading up to TRAP activation, it can be considered with a focus on targets after MAPK induction. Since various targets such as c-FOS, NFATc1, MITF, and TFE3 exist; thereafter, in this study, the inhibition of osteoclastogenesis mediators, such as c-Fos and NFATc1, was demonstrated as the main mechanism for inhibition of osteoclast differentiation by the OSE (Figure 4). Inhibition of osteoclast differentiation associated with NFATc1 has previously been reported as a treatment strategy for osteoporosis [30]. In addition, several studies have shown that inhibition of NFATc1 affects osteoblast differentiation by increasing the Fra-2 expression, which is helpful in anabolic therapy [31]. Further, previous studies have confirmed that five compounds in the OSE affect osteoblast differentiation, supporting the findings of this study [16].

Osteoporosis is often associated with degenerative arthritis and inflammatory reactions. An increase in the levels of cytokines due to an inflammatory response promotes osteoclast differentiation or inhibits osteoblast differentiation to advance osteoporosis [32]. Avenanthramides, a group of phenolic alkaloids that have only been reported in oat seedlings, have been shown to induce the NF-*κ*B inactivation in C2C12 cells and exhibit anti-inflammatory activity [33, 34]. In this study, the effect of the OSE on osteoclast differentiation was demonstrated *in vitro*. In the future, *in vivo* confirmation of the anti-inflammatory effect of the OSE and its synergistic effect on osteoclast or osteoblast differentiation will allow the emergence of the OSE as a strong candidate for osteoporosis treatment.

## 5. Conclusions

In this study, the OSE inhibited the RANKL-mediated osteoclasts. In particular, the formation of the TRAP + MNCs by the RANKL was attenuated, and the formation of anti-osteoclasts was confirmed by the OSE. The OSE also suppressed the formation of the TRAP + MNCs in the initial stage of osteoclast differentiation, but no significant results were observed in the middle or late stage. To elucidate how the OSE affects the signaling pathway, phosphorylation of JNK, ERK, P38, ATK, and MAP kinases, which are signaling pathway molecules in the early stages of differentiation, was stimulated, but no significant results were obtained. However, the expressions of c-Fos and NFATc1, which are essential in osteoclast differentiation, were dramatically suppressed by the OSE. Additionally, the mRNA expressions of OSCAR, DC-DTAMP, and cathepsin K were suppressed. In conclusion, the OSE-mediated inhibition of the RANKL-induced osteoclast differentiation is regulated by the suppression of the expression of c-Fos/NFATc1, a master factor in osteoclast formation, thus highlighting its effective application for the treatment of osteoporosis.

## Data Availability

All data generated or analyzed in this study are available in this article.

## Supplementary Materials

Supplementary Materials Supplementary Figure 1. Identification of extracts was performed by using the UPLC-CAD analysis system (DIONEX Ultimate 3000, Thermo scientific, Idstein, Germany). Analysis was performed by using the C18 analysis column (Thermo, AcclaimTM Polar Advantage II, C18, 4.6 × 250 mm, 5 *μ*m), and 0.1% formic acid in water (A), acetonitrile (B) (Sigma-Aldrich, MO, USA) was used as the mobile phase. The column oven was 35°C, the solvent flow was 1.0 ml/min, and the gradient was as follows: 0–3 min, 15% B; 9 min, 20% B; 16 min, 22% B; 20 min, 30% B; 36 min, 45% B; 38 min, 90% B; 40 min, 90% B; 42.1 min, 15% held for 9.9 min before returning to the initial conditions. Conventional methods were used for the identification and analysis of compounds, and standard retention times were compared for identification [18].
