# Supplementary material for: Oat Seedlings Extract Inhibits RANKL-Induced c-Fos/NFATc1 Transcription Factors in the Early Stage of Osteoclast Differentiation
Source: Evid Based Complement Alternat Med. 2022 Sep 23;2022:5372459. doi: 10.1155/2022/5372459 (PMC9525779; doi:10.1155/2022/5372459)
Supplement: Supplementary Materials — Supplementary Figure 1. Identification of extracts was performed by using the UPLC-CAD analysis system (DIONEX Ultimate 3000, Thermo scientific, Idstein, Germany). Analysis was performed by using the C18 analysis column (Thermo, AcclaimTM Polar Advantage II, C18, 4.6 × 250 mm, 5 μm), and 0.1% formic acid in water (A), acetonitrile (B) (Sigma-Aldrich, MO, USA) was used as the mobile phase. The column oven was 35°C, the solvent flow was 1.0 ml/min, and the gradient was as follows: 0–3 min, 15% B; 9 min, 20% B; 16 min, 22% B; 20 min, 30% B; 36 min, 45% B; 38 min, 90% B; 40 min, 90% B; 42.1 min, 15% held for 9.9 min before returning to the initial conditions. Conventional methods were used for the identification and analysis of compounds, and standard retention times were compared for identification [18]. [file 5372459.f1.docx]

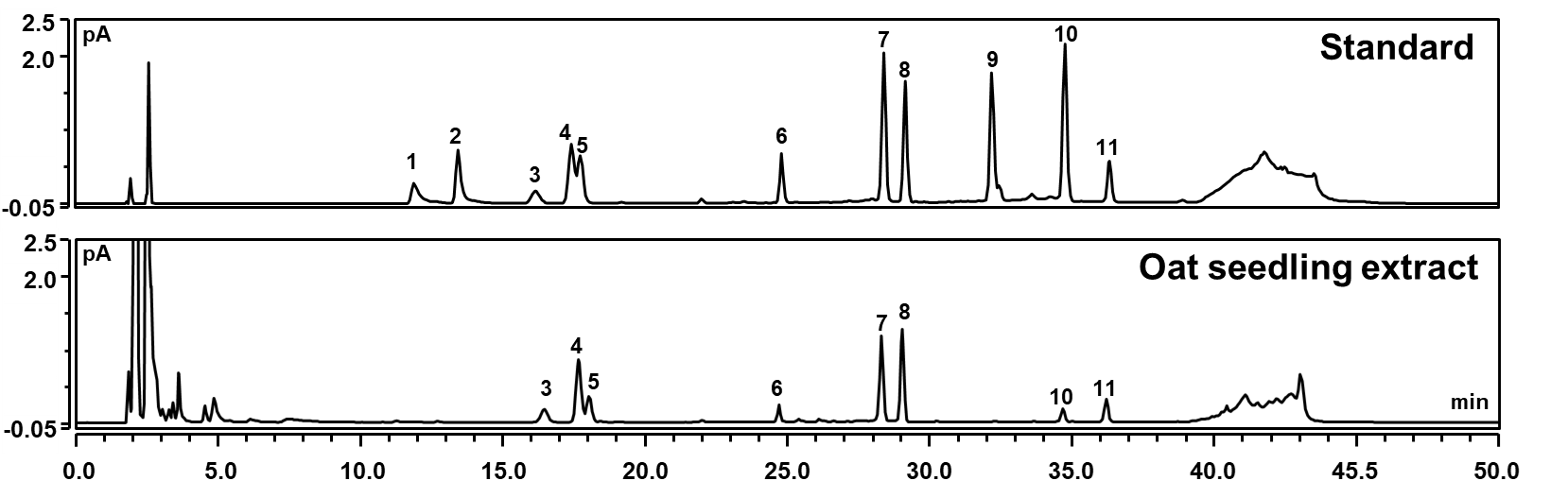


Supplementary Figure 1. UHPLC-CAD chromatogram of polyphenols in oat seedlings extract and standards. 3-O-feruloyl-quinic acid (**1**), isoorientin-6-β-d-xylopyranoside (**2**), vitexin-2″-O-rhamnoside (**3**), isoswertisin-2″-O-rhamnoside (**4**), isovitexin-2″-O-arabinoside (**5**), avenafuranol (**6**), avenacoside B (**7**), avenacoside A (**8**), diosgenoside (**9**), 26-desgluco-avenacoside B (**10)**, 26-desgluco-avenacoside A (**11**).
